# Obscuration Threshold Database Construction of Smoke Detectors for Various Combustibles

**DOI:** 10.3390/s20216272

**Published:** 2020-11-04

**Authors:** Hyo-Yeon Jang, Cheol-Hong Hwang

**Affiliations:** Department of Fire and Disaster Prevention, Daejeon University, 62 Daehak-ro, Dong-Gu, Daejeon 34520, Korea; fayahyo@gmail.com

**Keywords:** smoke detector, obscuration per meter (OPM), obscuration threshold, smoke color, performance-based fire safety design (PBD)

## Abstract

The obscuration thresholds for various smoke detectors and combustibles, required as an input parameter in fire simulation, were measured to predict the accurate activation time of detectors. One ionization detector and nine photoelectric detectors were selected. A fire detector evaluator, which can uniformly control the velocity and smoke concentration, was utilized. Filter paper, liquid fuels, and polymer pellets were employed as smoke-generation combustibles. The nominal obscuration thresholds of the considered detectors were 15 %/m, but the ionization detectors activated at approximately 40 %/m and 16 %/m, respectively, on applying filter paper and kerosene. In contrast, the reverse obscuration thresholds were found quantitatively according to the combustibles in the photoelectric detector. This phenomenon was caused by differences in the color of the smoke particles according to the combustibles, which is explained by single-scattering albedo (ratio of light scattering to light extinction). The obscuration thresholds for liquid fuels (kerosene, heptane and toluene) as well as fire types of polymer plastic pellets were also measured for several photoelectric detectors. A database of obscuration thresholds was thereby established according to the detector and combustible types, and it is expected to provide useful information for predicting more accurate detector activation time and required safe egress time (REST).

## 1. Introduction

To reduce the risk of fire due to the manhattanization, increased sizes and complexity of buildings, the number of countries introducing performance-based fire safety design (PBD) methods has been increasing [1]. The PBD approach generally assesses the fire risk based on a comparison between the available safe egress time (ASET) and the required safe egress time (RSET). This is based on a timeline analysis owing to the limitations of a complex review of the various factors that may affect the assessment. Specifically, the allowance of design uncertainties based on the ASET and RSET concepts can be expressed by a safety margin (ASET-RSET > safety margin) or a safety factor (ASET/RSET > safety factor). The RSET is evaluated as a relatively safe building from the impact of fire when it is larger than the ASET [2]. Therefore, to improve the reliability of fire risk assessment through PBD, the ASET and RSET must first be accurately calculated.

**Figure 1:**
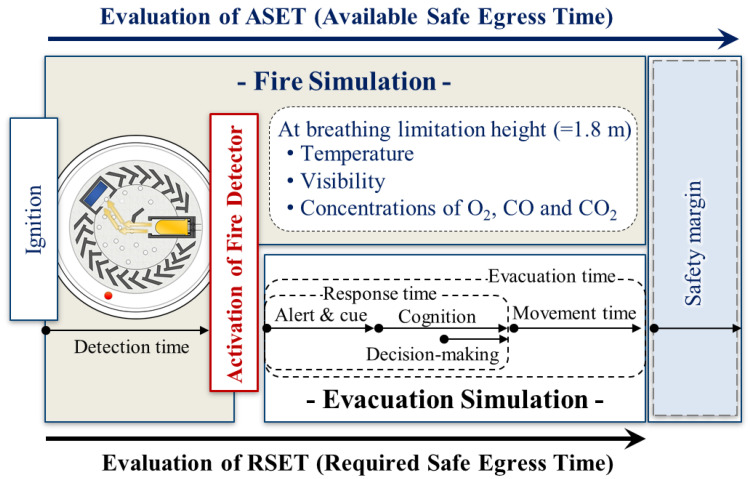
Classification of roles of fire and evacuation simulation for available safe egress time (ASET) and required safe egress time (RSET) evaluations.

The RSET, defined as the time it takes for the occupants in a building to evacuate to a safe place after the occurrence of a fire, is the sum of the fire detection time of the detector, the response time of the occupant (including the alarm and cue, recognition, and time delay to begin action), and the movement time [3]. The response time and movement time of occupants are evaluated by various evacuation simulations or theories [4,5]. Figure 1 shows a schematic diagram of the role of fire and evacuation simulations for ASET and RSET calculation. The detection time, which is the activation time of the smoke or heat detector after the occurrence of a fire, is included in the RSET calculation; however, it is predicted through the fire simulation [6]. As a result, the detection time prediction of the detector is directly related to the calculation of the RSET. It is affected by the prediction accuracy of the fire simulation based on various fire scenarios or fire conditions. In particular, the input parameters of the numerical model for smoke and heat detectors included in the fire models are important for accurately predicting the detection time of the detector, calculating the RSET, and finally securing the reliability of the fire risk assessment for the PBD. For reference, a fire dynamics simulator (FDS) [7], which can analyze fire behavior over time in a three-dimensional space, is widely used as a representative fire model for fire risk assessment.

The Heskestad and Cleary models have been applied to the FDS to predict the detection time of smoke detectors. These two numerical models can be classified as time lag methods in which a characteristic time (with a difference in smoke concentration existing between the housing and the sensing chamber), takes into account the entry resistance of smoke due to the shape of the inlet of the smoke detector [8]. In the Heskestad model [9,10], the delay time (or characteristic time) is the time required for the smoke to reach from outside of the detector to the inside. It is expressed as the ratio of the characteristic length (L, m) and the free stream velocity (U, m/s) [11,12], where L is an input parameter that must be determined experimentally. In the Cleary model [13], the mixing time inside the detector is also considered. It is the total delay time expressed by the sum of the dwell time (δt) required to flow into the sensing chamber from the outside and the mixing time (τ) inside. The smoke flow is divided into the plug flow and perfectly stirred flow. δt and τ are expressed as functions of U, and additional input parameters ($\alpha_{e}$, $\beta_{e}$, $\alpha_{c}$, $\beta_{c}),$ which must be determined experimentally, are required. In other words, the input parameters for expressing the time lag of the detector activation differ according to the applied numerical model.

On the other hand, as a common input parameter to both the Heskestad and Cleary models, the obscuration threshold value—which is the obscuration per meter (OPM) at the moment the detector is activated—is required. The OPM is defined by Equation (1) and is signified by the ratio (%) of the intensity of light extinction by smoke particles per unit length (m).

(1) $$\mathrm{OPM}\;=\;\left(1-{\left(I/I_{o}\right)}^{1/L_{p}}\right)\;\times 100\;\left(\%/m\right)$$

where $I/I_{0}$ is the ratio of the intensity of light extinction by smoke particles, and $L_{p}$ is the light path length (m). The detailed concept and measurement methods of the individual and common input parameters required in the Heskestad and Cleary models can be found in previous studies [14,15].

Recently, our research team developed a test device—a fire detector evaluator (FDE)—to measure the input parameters required in the numerical model of smoke detectors. The FDS-based smoke detector input parameter values that are widely applied to PBD were presented [16]. Specifically, the input parameters of the Heskestad [9,10] and Cleary [13] numerical models that could predict the activation of specific photoelectric and ionization smoke detectors were measured. The fire simulation results that applied the input parameters obtained through the experiment correctly predicted the activation time of the detector measured under the same fire conditions, whereas large errors in the detector activation times occurred when the input parameters suggested in the FDS User’s Guide were applied [14,15]. In addition, through sensitivity analysis of the input parameters required in the smoke detector numerical model, it was found that the obscuration threshold values, which are common parameters, had the greatest influence on detector activation compared to the development factors required in each numerical model [17]. Considering smoke detectors with a wide variety of structural characteristics and various types of combustibles, the results of the present study not only highlight the importance of measuring the input parameters of smoke detector numerical models but also provide information on important input parameters that must be initially considered.

In general, quantitatively accurate values were not provided for the obscuration thresholds of detectors. Only the application range or nominal values according to detector sensitivity tests were provided by the manufacturers. Therefore, in previous studies that predicted detector activation times using fire simulation, precise obscuration threshold measurements or additional verification processes were not specifically considered [18,19,20]. Furthermore, although the changes in smoke color due to the combustibles can produce a significant change in the light scattering signals applied to smoke detectors, the quantitative difference of the obscuration thresholds according to various combustibles has not yet been investigated.

For the ultimate purpose of improving the prediction accuracy of the detector activation time by constructing a database (DB) of input parameters required in the fire simulation for various smoke detectors and combustibles, measurement of the detector obscuration thresholds and DB construction was performed in this study. To this end, a total of ten detectors, mainly installed in South Korea, were reviewed. Firstly, the quantitative differences in the obscuration thresholds of the ionization and photoelectric detectors according to the colors of the smoke particles were examined. In addition, filter paper, various liquid fuels, and polymer pellets were considered as the combustibles. The differences in obscuration threshold values, which were based on the combustibles in photoelectric detectors to which the light scattering method was applied, were assessed. Performing the PBD through the DB provided in this study is expected to enable prediction of the activation time of detectors far more accurately by applying the appropriate input parameters according to the combustible material.

## 2. Experimental Conditions

### 2.1. Experimental Method and Procedures

**Figure 2:**
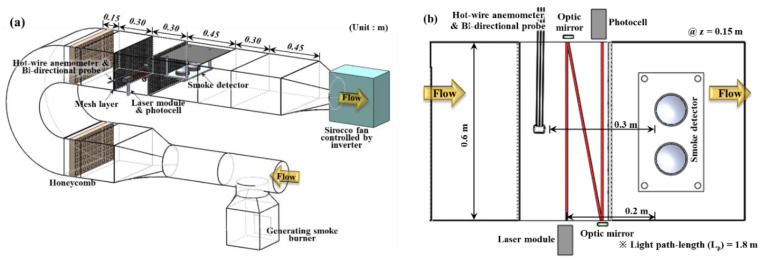
Schematics of (**a**) Fire Detector Evaluator (FDE) and (**b**) test section for measuring input parameters required in the numerical models of a smoke detector [16].

To measure the obscuration threshold—a common input parameter of smoke detector numerical models—an FDE was used, as shown in Figure 2 [16]. Figure 2a shows the overall shape of the FDE, which was made with a square carbon steel duct with a cross-sectional area of 0.18 m^2^ (0.6 m × 0.3 m). It was fastened with rubber packing. The inside of the duct of the FDE was designed to realize a uniform smoke flow over time and space. To reduce the intensity of turbulence, a smoke generator was placed in the front and a sirocco fan was placed in the rear of the FDE based on the detector and the measurement locations [21]. The flow rate was controlled using the sirocco fan, and the damper was controlled by means of an inverter. In addition, a honeycomb and mesh were installed to form a uniform smoke flow. As a smoke-generating device installed at the bottom of the FDE, a burner (optimized according to the type of combustibles) was installed to enable continuous smoke generation.

Figure 2b shows the measurement location of the velocity and smoke concentration located at the center of the duct at a vertical height of z = 0.15 m. The velocity was measured using a bi-directional probe calibrated by a hot wire anemometer (Testo 480) to accurately measure the low-speed flow and reproducibility of the experiment. The obscuration threshold for the detector was measured using a light-extinction method [22], detailed device descriptions can be confirmed in previous studies [23]. To minimize measurement errors of the OPM due to forward scattering, approximately 1.8 m of $L_{p}$ between the laser module (650 nm) and the photocell was applied. Details of $L_{p}$ selection and the FDE can be found in previous studies [16]. The power of the smoke detector was supplied from a p-type fire control panel, and the voltage signal to check the smoke concentration and the activation of the detector were recorded in real time at 1-s intervals using a DAQ (Graphtec, GL 820). 

**Figure 3:**
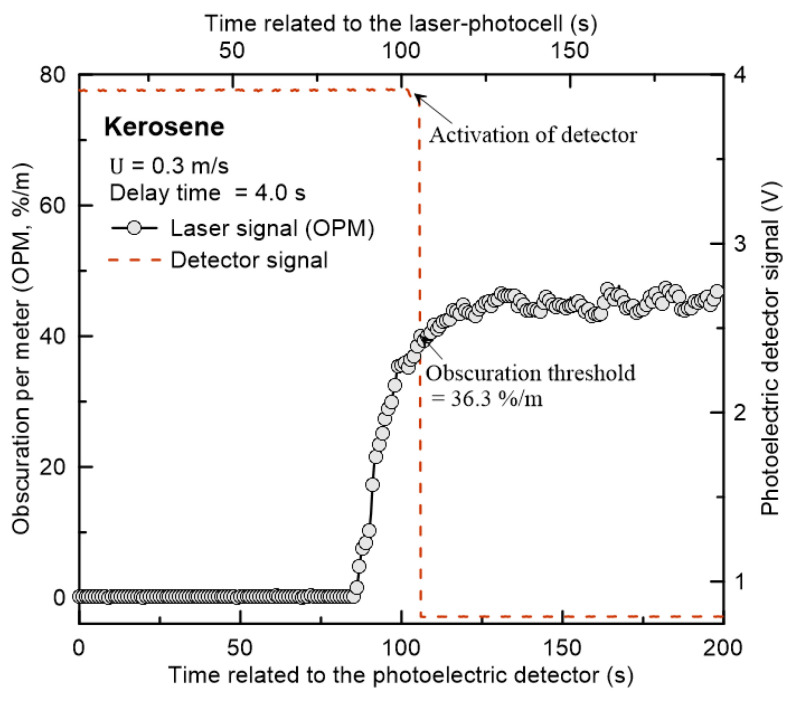
Procedure for measuring the obscuration threshold of smoke detector using FDE.

The measurement process of the obscuration threshold value, an input parameter that has the greatest influence on the detector activation time, is explained in Figure 3. The results in the figure show the voltage signals associated with the activation of the OPM and the detector over time after kerosene ignition using a lamp wick. In the detector sensitivity test, considering that the smoke flow velocity was 0.2–0.4 m/s [24], the average velocity (U) inside the FDE was fixed at 0.3 m/s. The delay time, which is the time at which the smoke OPM was measured to the moment the smoke flowed into the detector (based on the moment when the voltage increased in the detector’s internal circuits), was 4.0 s. The OPM of the smoke reaching the test section inside the FDE increased relatively linearly with time and reached a quasi-steady state at approximately 55 %/m. The voltage signal associated with the detector’s activation showed that the concentration of the smoke gradually increased, and a voltage drop occurred when it exceeded the obscuration threshold. In other words, the OPM was synchronized over time and the voltage signal related to the detector activation had a delay time of 4.0 s. Consequently, the obscuration threshold value of 36.3 %/m, which was the OPM at the moment the detector was activated, could be obtained. Obscuration threshold measurements were repeated at least five times under the same experimental conditions, and the average value and standard deviation expressed in the form of a vertical error bar are shown in the graph.

### 2.2. Selection of Smoke Detectors and Smoke Generators for Various Combustibles

Smoke detectors have a wide variety of structures, determined by the shape of the housing according to the manufacturer, or the principle of fire detection, the structure of the optical maze to form the dark room inside the sensing chamber, the porosity of the mesh to prevent the inflow of foreign substances, and the angles of the light-emitting and the light-receiving parts. Therefore, considering the efficiency of the DB construction for the obscuration thresholds of the detectors, one ionization and nine photoelectric smoke detectors were selected. All smoke detectors reviewed in this study were of the two-class kind, and the nominal obscuration threshold was 15 %/m based on the information provided by the manufacturers [24]. The photoelectric detectors were named A through I for convenience. They were selected through the investigation of a number of installations in South Korea over the past three years and by leveraging the expertise of approximately 30 related specialists. In practice, even if the same detector is applied, a difference in the obscuration threshold may occur owing to the differences in light scattering and light-extinction characteristics of the smoke particles according to the combustibles. Therefore, various combustibles, such as filter paper, liquid fuels, and polymer pellets, were considered.

**Figure 4:**
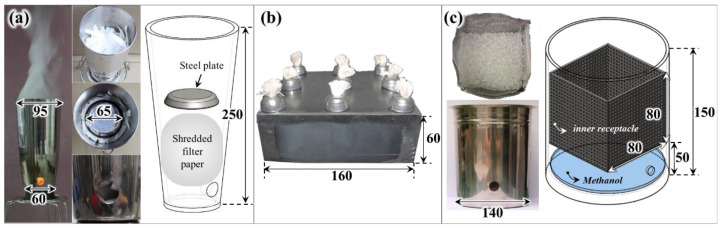
Photographs and schematics of smoke generators using (**a**) filter paper, (**b**) liquid fuel and (**c**) polymer pellets (unit:mm).

Figure 4 shows a photograph and schematic diagram of a smoke-generating device based on the type of combustible material. Different shapes of burners were applied according to the combustibles to enable control of the amount of smoke generated. Filter paper, produced by ADVANTEC, is a standard combustible applied to the sensitivity test of detectors in South Korea [24]. In general, to generate white smoke from filter paper, smoldering combustion is induced by placing it on a 400 °C hot plate or by cutting a long piece of paper and burning it in a receptacle (or burner) [25,26,27]. However, it is difficult to control a uniform amount of smoke over time, and it was found that the high-temperature smoke flow due to intermittent flaming combustion caused thermal stratification inside the FDE. Since the occurrence of thermal stratification causes fluctuations in smoke concentration and flow velocity inside the FDE, it can lead to different detection times and low measurement reproducibility, depending on the vertical height of the detector [28]. Therefore, in this study, 20 g of shredded filter paper (approximately 10 × 40 mm) was irregularly stacked into a receptacle. Then, a stainless-steel plate with a 65-mm diameter was installed on the top, as shown in Figure 4a. When the paper was ignited through a hole located at the bottom of the receptacle, the steel plate moved to the bottom very slowly. As a result, the occurrence of flaming combustion due to sufficient air contact of the paper was suppressed, and a sufficient amount of smoke at a very low temperature was generated for the duration of the experiment. 

In general, a pool fire—which can easily control the scale of a fire owing to changes in the surface area of the fire source—is applied to liquid fuels that generate black smoke; however, high-temperature smoke flow due to the flame can cause significant thermal stratification. Therefore, in this study, a cuboid burner with an inserted lamp wick, as shown in Figure 4b, was applied to minimize the heat generated and to control the amount of smoke generated [16]. Smoke generation using a wick has the advantage that it is easy to control the amount of smoke produced through changes in the diameter, exposed length, and the number of wicks. Kerosene (C_12_H_24_), heptane (C_7_H_16_), and toluene (C_7_H_8_)—with a relatively high soot tendency—were considered the liquid fuels, and the number of wicks was changed according to each liquid fuel to generate smoke that could reach the obscuration threshold of the detector.

Figure 4c shows a photo and schematic diagram of a smoke generator of polymer plastics that can be easily identified as combustibles in modern buildings. For ease of ignition and continuous burning of solid combustibles, pellets of approximately 5 mm in diameter were applied. For initial ignition, 10 mL of methanol was supplied to the bottom of the receptacle, and 2 g of pellets were supplied to the inner receptacle of an 80 mm long cube made of steel mesh. Methanol flame can supply sufficient heat for the pyrolysis of the pellets; however, it may not have a significant effect on the obscuration threshold (on account of the burning of polymers) because of the small quantity of soot generated. Thus, since the supply of methanol and pellets was very small in this study, thermal stratification did not occur in the FDE. This was clearly observed through the uniformity of the smoke concentration along the vertical height in the test section.

**Figure 5:**
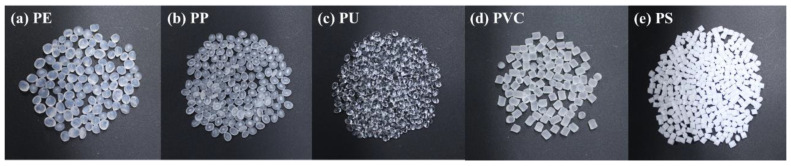
Photographs of polymer pellets (**a**) polyethylene, (**b**) polypropylene, (**c**) polyurethane, (**d**) polyvinyl chloride and (**e**) polystyrene.

Figure 5 shows photographs of the polymer pellets—polyethylene (PE), polypropylene (PP), polyurethane (PU), polyvinyl chloride (PVC), and polystyrene (PS). These polymers are representative components of combustibles, such as mattresses, sofas, and home appliances, which are common in building fire scenarios.

## 3. Results and Discussion

### 3.1. Obscuration Threshold of Ionization and Photoelectric Detectors according to Smoke Particle Colors

**Figure 6:**
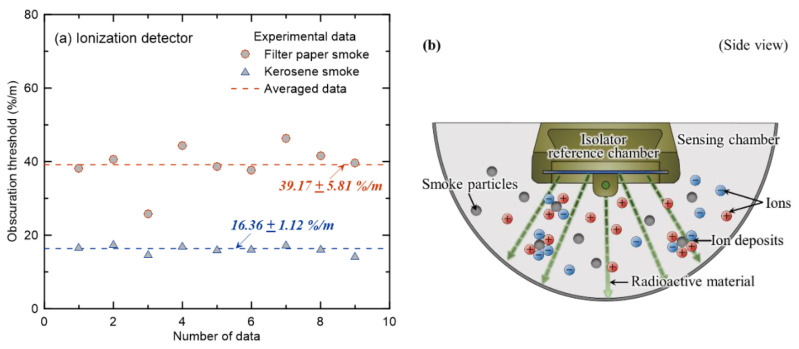
For the ionization smoke detector, (**a**) obscuration thresholds with filter paper and kerosene and (**b**) the conceptual diagram of the detector.

**Figure 7:**
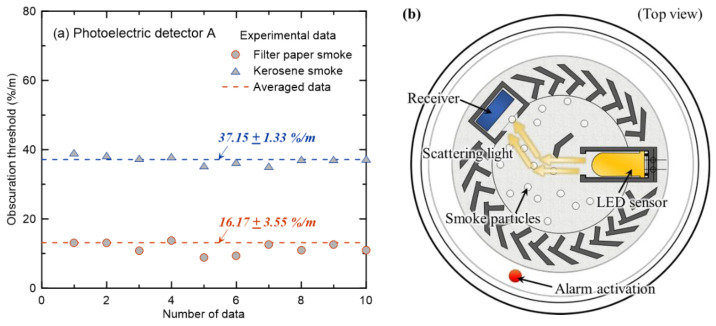
For the photoelectric smoke detector, (**a**) obscuration thresholds with filter paper and kerosene and (**b**) the conceptual diagram of the detector.

Smoke detectors can generally be classified into ionization-type and photoelectric-type detectors based on the detection method. Figure 6 and Figure 7 show the obscuration thresholds of ionization and photoelectric detectors for filter paper and kerosene, in which the smoke particles are white and black, respectively. The measurement of the obscuration threshold for each combustible material with the same detector was repeated ten times, as shown in Figure 3. A new detector was then installed that had not been used in any preceding experiment.

Firstly, the results of the ionization-type detector in Figure 6a show that the average obscuration threshold obtained from repeated experiments is 39.17 %/m, and the standard deviation is 5.81 %/m when filter paper is applied. The average obscuration threshold of kerosene is 16.36 ± 1.12 %/m, showing a significant difference compared to that of the filter paper. This can be explained by the structure and principle of the ionization detector shown in Figure 6b. The structure of the ionization detector is divided into a sensing chamber (external ion chamber) and an isolator reference chamber (internal ion chamber). The air in the sensing chamber is ionized by a small amount of radioactive material emitted from the isolator reference chamber. When smoke-containing soot generated from a fire enters the sensing chamber, it deposits ions and increases the electrical resistance inside the sensing chamber. In other words, when the flow of electric current due to the inflow of smoke particles decreases below a set value, the detector is activated. Water vapor accounts for the largest proportion of white smoke generated from filter paper, and black smoke generated from kerosene accounts for a substantial proportion of soot generated by the bonding and growth of carbon particles. Consequently, it can be expected that the detector activates at a relatively low OPM as the soot in the black smoke deposits ions more easily compared to the water vapor in white smoke [16].

Figure 7a shows the results of photoelectric detector A under the same combustibles and experimental conditions as shown in Figure 6. On average, the obscuration threshold of smoke generated by the smoldering combustion of filter paper is 16.17 ± 3.55 %/m, and the obscuration threshold was measured to be 37.15 ± 1.33 %/m under the flaming combustion conditions of kerosene. In other words, contrary to the ionization detector results, the detector was activated at a relatively high OPM under the flaming combustion conditions of kerosene, which generated black smoke. Although the principles of smoke detection by ionization and photoelectric detectors are different, it was interesting to discover that the obscuration threshold had the opposite quantitative result based on the colors of the smoke particles. To analyze the cause of the above phenomenon, the structure and principle of the photoelectric detector were assessed. As shown in Figure 7b, the detector was activated when a certain amount of scattered light (incident light from the LED sensor, which was scattered by smoke particles) reached the receiver (photocell). Based on the incident light extinction through the scattering and absorption by smoke particles, black smoke is expected to have less light extinction through scattering than white smoke. This phenomenon can be clearly explained through a review of single-scattering albedo (SSA) [29], which is expressed as the ratio of light scattering to light extinction (sum of scattering and absorption). The SSA factor was studied to examine the scattering characteristics of soot according to the intrinsic optical properties of smoke generated from various fuels. If the SSA = 1, it means that the light extinction of smoke particles is only due to scattering, whereas if the SSA = 0, it means that light extinction is caused primarily by absorption of smoke particles. Specifically, black smoke particles (generated from kerosene) have been reported as having an SSA = 0.3 [29] at a wavelength of 532 nm, and white smoke particles (mainly generated from paper or wood fires) have been reported as having an SSA = 0.6~0.8 [30] at a wavelength of 500 nm. This is why the detector activated at a higher OPM when kerosene was applied than when filter paper was used.

Summarizing the results of Figure 6 and Figure 7, quantitatively similar obscuration thresholds were found under the conditions of white smoke in the ionization detector and black smoke in the photoelectric detector owing to the differences in the activating principles according to the detector type. The value was very high at approximately 40 %/m. The smoke detectors reviewed in this study were of the two-class types [24] with a nominal obscuration threshold of 15 %/m. Thus, to accurately predict the detector activation time, an additional review of various combustibles with different smoke particle colors had to be considered carefully in addition to the detector type.

### 3.2. Database Construction of the Obscuration Thresholds with Liquid Fuels and Polymer Pellets for Various Models of the Photoelectric Smoke Detector

As mentioned above, the nominal obscuration threshold for several types of photoelectric smoke detectors applied in the activation and non-activation tests for the performance test of smoke detectors was equally implemented at 15 %/m based on the two-class type [24]. However, activation of the photoelectric detector was determined by the correlation among the smoke characteristics, smoke transport, and detector characteristics. Therefore, to consider the optical properties of the smoke particles, a review of various fuels was required. In addition, because there was a difference in various structures and sensitivity inside the sensing chambers, depending on the detector manufacturer, it was necessary to calculate an accurate obscuration threshold for several detectors with a high frequency of use. Since ionization detectors containing radioactive substances have high costs of disposal after their useful lifetimes, their production and usage are very low compared to those of photoelectric detectors. Therefore, only photoelectric detectors were considered for the examination of the obscuration threshold based on different combustibles.

**Figure 8:**
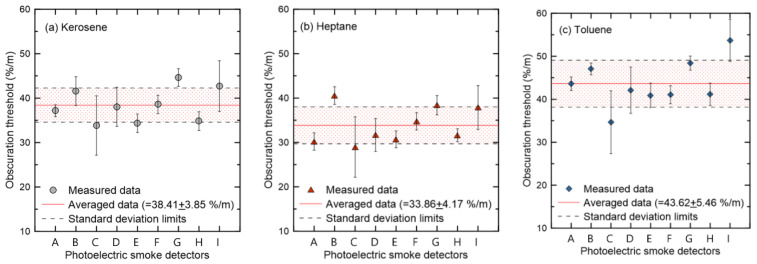
Comparison of obscuration thresholds with liquid fuels ((**a**) kerosene, (**b**) heptane and (**c**) toluene) for various models of the photoelectric detector (A~I).

Figure 8 shows the obscuration threshold when the liquid fuels kerosene, heptane, and toluene were applied as the combustibles targeting photoelectric detectors (A–I) from the different manufacturing companies. Five replicates were performed on the same detector models, and the symbol and vertical error bars in Figure 8 represent the mean and the standard deviation of each, respectively. Figure 8a shows the result of kerosene, and it was found that even if the same detector model was used, the standard deviation of the obscuration threshold varied considerably depending on the manufacturer. The average obscuration threshold of each detector model was also significantly different. The average value for all detectors was 38.41 %/m, showing a standard deviation of 3.85 %/m. In other words, even if the same experimental conditions and combustibles were considered, the obscuration threshold had a quantitative difference that could not be ignored, not only between the detector models of different manufacturers but also between detectors from the same manufacturer. Nevertheless, the provision of an average obscuration threshold for a large number of detectors with a high frequency of use can have a significant meaning for PBDs that evaluate fire risk using fire simulation. The detector model of a specific manufacturer is not determined specifically in the PBD process, and obscuration thresholds, which are common input parameters of the detector model for fire simulation, are arbitrarily set by the user. Therefore, the application of the average obscuration threshold measured for multiple detectors is an appropriate approach for a more accurate detector activation time and RSET calculation from a statistical point of view.

As shown in Figure 8b,c with heptane and toluene applied, the average obscuration thresholds of the considered detectors were 33.86 and 43.62 %/m, respectively. As shown in Figure 7, it was found that the detector activated at a smoke concentration much higher than the nominal obscuration threshold of 15 %/m for these black-smoke-generating liquid fuels. In terms of the quantitative differences in the obscuration threshold of the considered liquid fuels, the highest values are observed in the order of toluene–kerosene–heptane. The cause can be analyzed by comparing the SSA and mass specific extinction coefficient ($K_{m}$) [31,32]. To this end, a detailed comparative review of the optical properties of smoke particles from each fuel should be performed. A direct comparison of the values provided in the literature has a clear limit owing to the quantitative difference between the SSA and $K_{m}$ (the wavelength of incident light). Therefore, an investigation of the differences in the obscuration threshold for liquid fuels that generate visually similar black smoke particles was not considered in the scope of this study.

**Figure 9:**
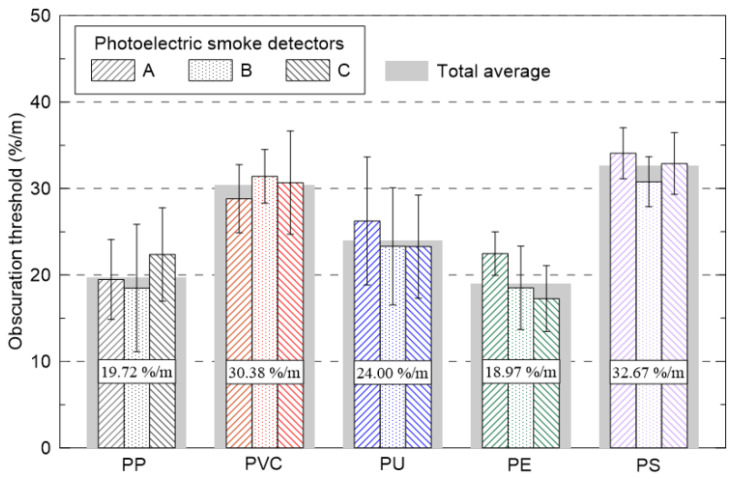
Comparison of obscuration thresholds with various polymer pellets (polypropylene (PP), polyvinyl chloride (PVC), polyurethane (PU), polyethylene (PE) and polystyrene (PS)) for the photoelectric detector models (A–C).

Figure 9 shows the obscuration thresholds of photoelectric detectors A–C when five types of polymer plastic (PP, PVC, PU, PE, and PS) pellets, which can be easily identified as combustibles in modern buildings, were applied. The average values of the three manufacturers’ detectors based on the composition of the polymer pellets are simultaneously shown as shades and numerical values in the figure. The obscuration threshold of the polymer pellets differed significantly, from a minimum of approximately 19 %/m to a maximum of approximately 33 %/m, depending on the pellets. The obscuration thresholds of PP and PE were 19.72 and 18.97 %/m, respectively, showing quick response characteristics regardless of the detector model. On the other hand, the obscuration thresholds of PVC and PS were 30.38 and 32.67 %/m, respectively. It was expected that the detector activation could be slower under the same assumed fire conditions. In Figure 8, the differences in the obscuration thresholds of the polymer plastics according to their respective compositions are shown. Detailed analysis is difficult owing to limited data on the SSA and $K_{m}$. However, a schematic analysis of the differences among them, depending on the colors of the smoke particles, may be possible through the analytical method shown in Figure 7.

**Figure 10:**
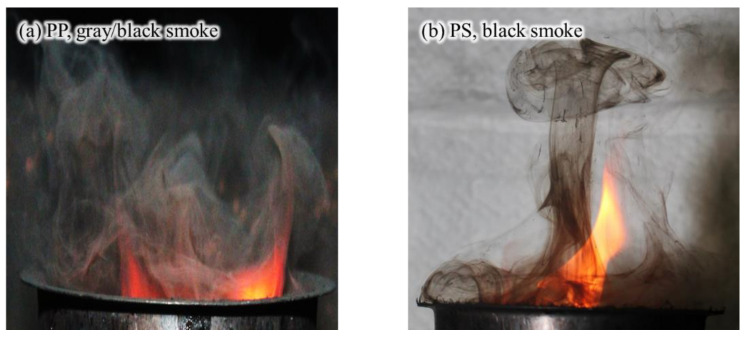
Photographs of smoke flow generated from burning of (**a**) PP and (**b**) PS pellets.

Figure 10 shows an instantaneous picture of the flame and smoke generated when the PP and PS pellets were burned under the same ventilation conditions. When the PP pellets were burned, white smoke particles were generated, and the flames were visually gray and black. On the other hand, PS pellets generated a large quantity of soot, and black smoke was clearly identified. Although the color of smoke cannot be clearly defined visually, gray and black smoke was visible in the PP and PE samples, and black smoke was visible in the PVC and PS samples. As a result, it was expected that the PP and PE containing white smoke particles would have a lower obscuration threshold owing to the higher ratio of scattered light in the extinction light. However, it was presumed that the PVC and PS samples, in which light extinction was mainly caused by absorption, would have a relatively high obscuration threshold.

Table 1 summarizes the obscuration thresholds of filter paper, liquid fuels, and polymer pellets for ionization and photoelectric detectors, respectively, as shown in Figure 6, Figure 7, Figure 8 and Figure 9. The obscuration threshold of the proposed ionization detector is an average value that was obtained by repeating the tests five times for the same detector model. In addition to the repeated experiments, the average value of the nine photoelectric detectors was simultaneously measured. As a result, the measured obscuration thresholds presented for various combustibles could be assessed to have had sufficient reliability (from a statistical point of view) to be used as an important input DB for predicting the activating time of a detector using fire simulations. In addition, more specific device properties of detectors required as input parameters for fire simulations can be found on the Fire Technology Solution DB website, which is openly activated for engineers performing PBD [33].

## 4. Conclusions

The objective of this study was to improve the prediction accuracy of the detector activation time by establishing a DB of input parameters required for the fire simulation of various smoke detectors and combustibles. The obscuration threshold, which is the OPM (%/m) at the moment the detector is activated, was measured. To this end, one ionization detector and nine photoelectric detectors, which are frequently used in South Korea, were selected. In addition, a fire detector evaluator (FDE) that could uniformly control the velocity and smoke concentration was used. Filter paper, liquid fuels, and polymer pellets were employed as the combustibles for smoke generation. The main results are described below.

The nominal obscuration thresholds of the considered smoke detectors were all 15 %/m; however, the ionization detectors operated at approximately 40 and 16 %/m, respectively, when the filter paper and kerosene were applied. The reverse obscuration thresholds were quantitatively determined according to the combustibles in the photoelectric detector. This phenomenon was caused by the differences in smoke particles, which were white and black, based on the combustibles. It was specifically explained through the SSA, which is defined as the ratio of light scattering to light extinction.

The average obscuration thresholds of the liquid fuels, such as kerosene, heptane, and toluene, were measured for nine photoelectric detectors. Even under the same experimental conditions and combustibles, significant differences in obscuration thresholds were found, not only between the detectors of different manufacturers but also between the detectors of the same manufacturer. As a result, the application of the average obscuration threshold measured for multiple detectors can be deemed an approach to predicting a more efficient detector activation time at the PBD stage where the detector model has not yet been determined.

The obscuration threshold of the photoelectric detector was measured for five types of polymer plastic (PP, PVC, PU, PE, and PS) pellets that can be easily identified as combustibles in modern buildings. The lower obscuration thresholds of PP and PE compared to those of PVC and PS were analyzed through the visualization of gray and black smoke particles, including white smoke.

In conclusion, a DB was constructed (based on the detector type and combustible type) of the obscuration threshold required as the detector input information for a fire simulation. The results of this study are expected to provide useful information for achieving more accurate detector activation times and RSET predictions. More specific effects of smoke particle characteristics according to ventilation and burning type such as flaming and flameless (smoldering) combustion on the obscuration threshold of smoke detector will be carried out in future studies.

## Figures and Tables

**Table 1:** Summary of obscuration thresholds (%/m) of filter paper, liquid fuels and polymer pellets (for the photoelectric smoke detector, the average value of all considered detectors is presented).

	Filter Paper	Kerosene	Heptane	Toluene	PP	PVC	PU	PE	PS
Ionization detector	39.2	16.4	11.1	-	-	-	-	-	-
Photoelectric detector	16.2	38.4	33.9	43.6	19.7	30.4	24.0	19.0	32.7

## Abbreviations

The following abbreviations are used in the manuscript:

PBD	Performance-Based Fire Safety Design	L_p_	Light Path Length [m]
ASET	Available safe egress time	FDE	Fire detector evaluator
RSET	Required safe egress time	DB	database
FDS	Fire dynamics simulator	PE	Polyethylene
L	Characteristic length [m]	PP	Polypropylene
U	Free stream velocity [m/s]	PU	Polyurethane
δt	Dwell time	PVC	Polyvinyl chloride
τ	Mixing time	PS	Polystyrene
$\alpha_{e}$, $\beta_{e}$, $\alpha_{c}$, $\beta_{c}$	Additional input parameters	SSA	Single scattering albedo
OPM	Obscuration per meter [%/m]	K_m_	Mass specific extinction coefficient [m^2^/g]
